# Supplementary material for: DNA methylation patterns identify subgroups of pancreatic neuroendocrine tumors with clinical association
Source: Commun Biol. 2021 Feb 3;4:155. doi: 10.1038/s42003-020-01469-0 (PMC7859232; doi:10.1038/s42003-020-01469-0)
Supplement: Supplementary file 1 — Supplementary Information [file 42003_2020_1469_MOESM1_ESM.pdf]

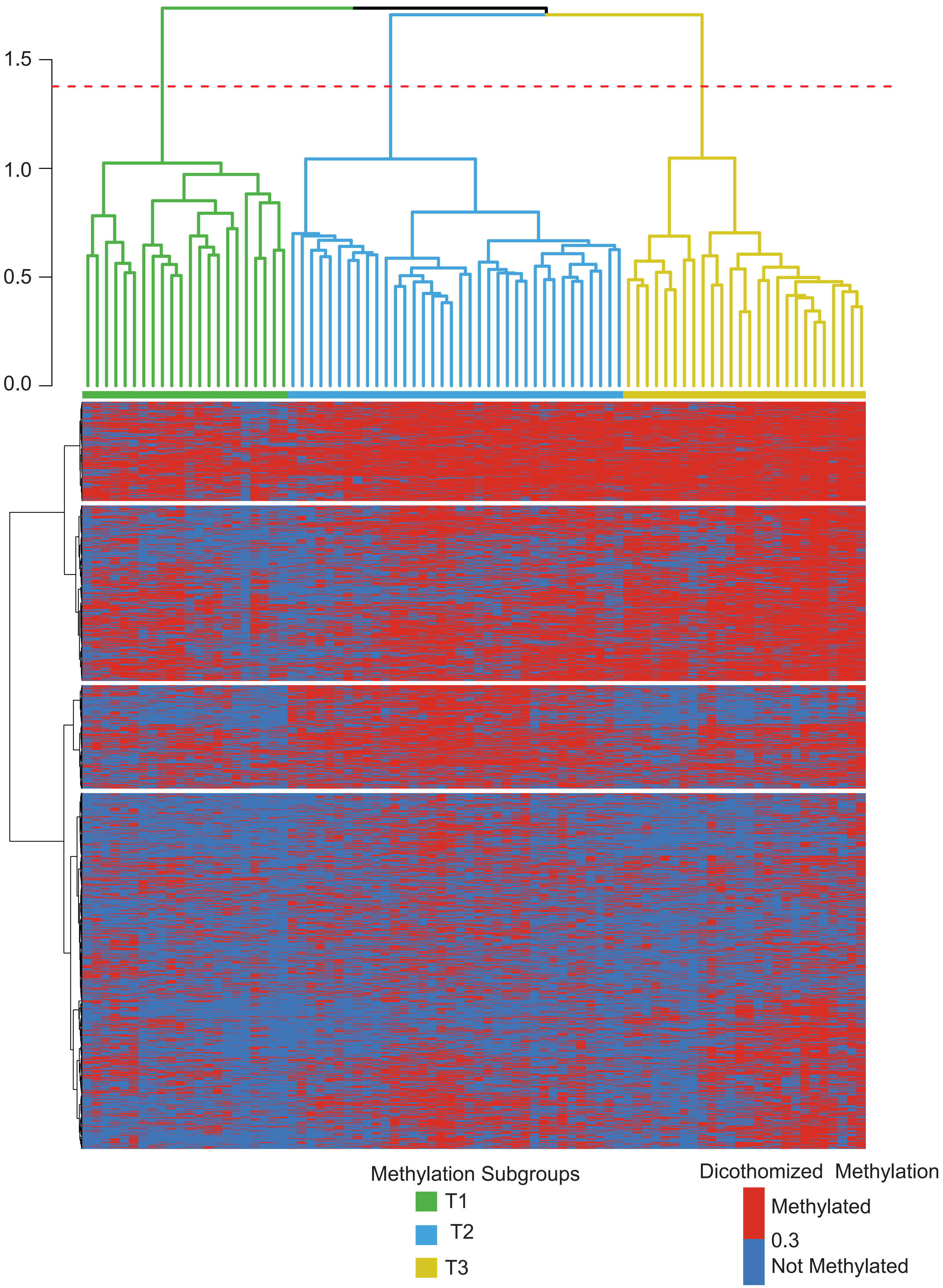

Supplementary Fig.1: Three subgroups identified based on methylation values.
A total of 3,378 CpG sites located in promoter regions that were not methylated across all normal pancreata samples and had a standard deviation >0.20 of the DNA methylation levels across 84 tumors. The methylation levels of these 3,378 most variable CpG sites were dichotomised (representing a presence of methylation; beta value ≥0.3, or absence of methylation: beta value <0.3 of methylation), and clustered to obtain potential subgroups of tumor samples. The unsupervised clustering used a binary distance measure and Ward's clustering method.

a

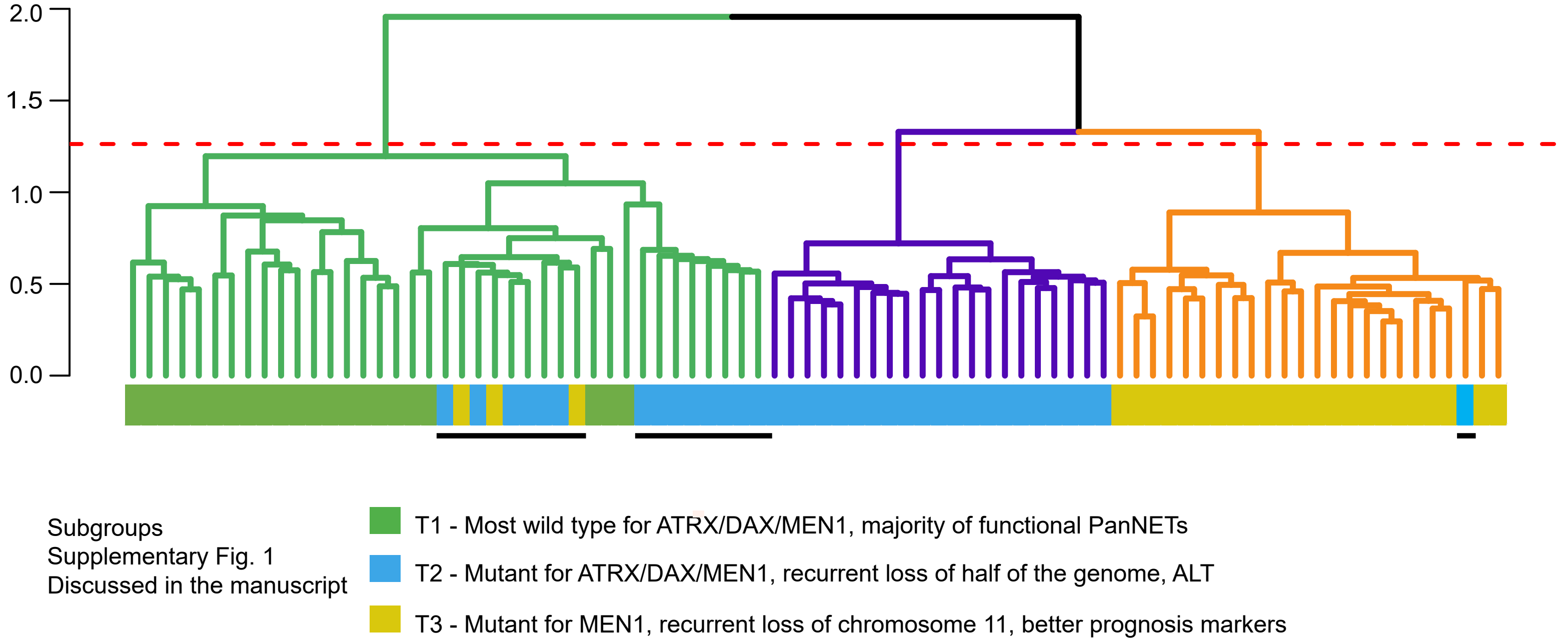

b

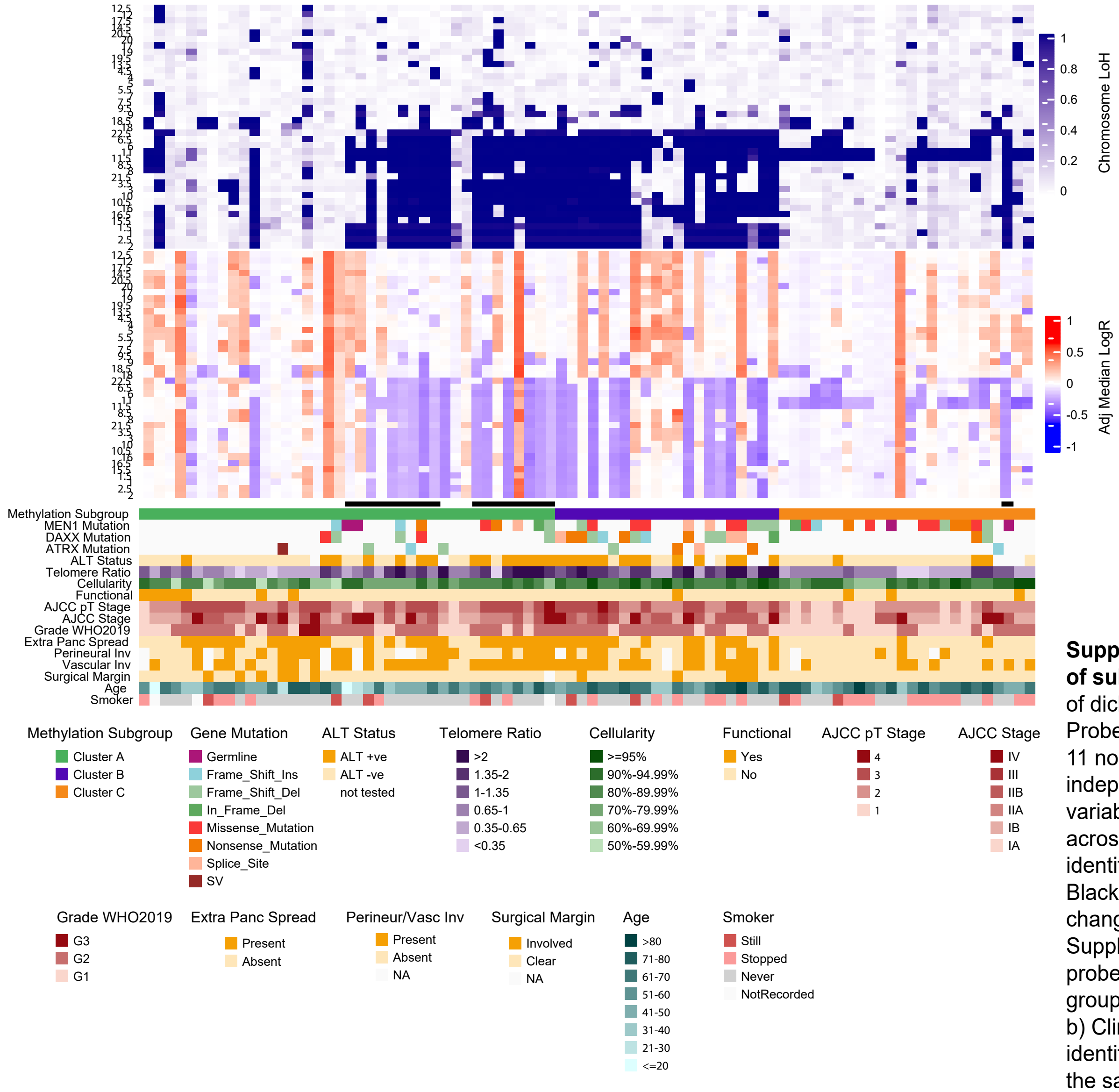

Supplementary Fig. 2: Identification of subgroups. a)Unsupervised clustering of dichotomized methylation of 7,227 probes. Probes were selected if not methylated across 11 normal adjacent pancreata (<0.30, independent of genomic location) and most variable probes across 84 tumors (SD>0.20) across the entire genome. Three clusters were identified A (green), B (purple) and C (orange). Black underscore lines show samples that changed subgroups from the clustering in Suplemantary Fig.1 using only promoter probes. Colours in the bars show the groups presented in Supplementary Fig.1. b) Clinical features of the 3 sub-groups identified in a. Tumors are presented in the same order of the clustering (a). Black underscore lines show samples that changed subgroups from the original analysis presented in Supplementary Fig.1.

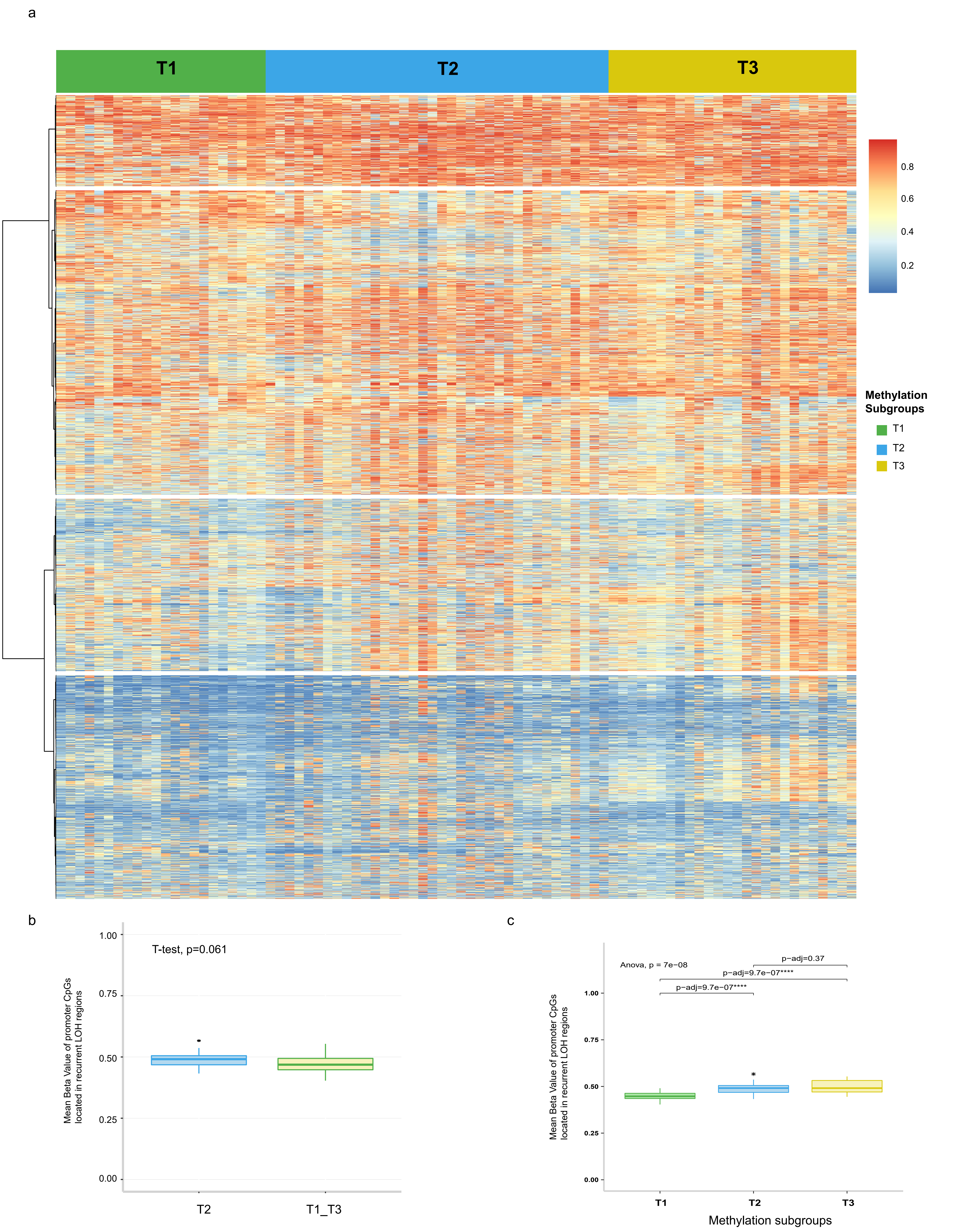

Supplementary Fig. 3: Gene promoter methylation across recurrent LOH regions. a) Heatmap of methylation levels of 6,861 most variable CpG sites (SD≥0.20 across tumors) located within promoter regions in chromosomes that presented recurrent LOH. b) Methylation levels do not suggest loss of methylation in subgroup T2 (n=36) compared to the other two subgroups together (T1 and T3, n=48; p value =0.061; T test). c) When comparing the overall level of methylation across the three subgroups T1 (n=22) presented lower levels of methylation (adjusted p value=9.7e-07) than T2 (n=36), which includes tumors with recurrent LOH of half of the genome.

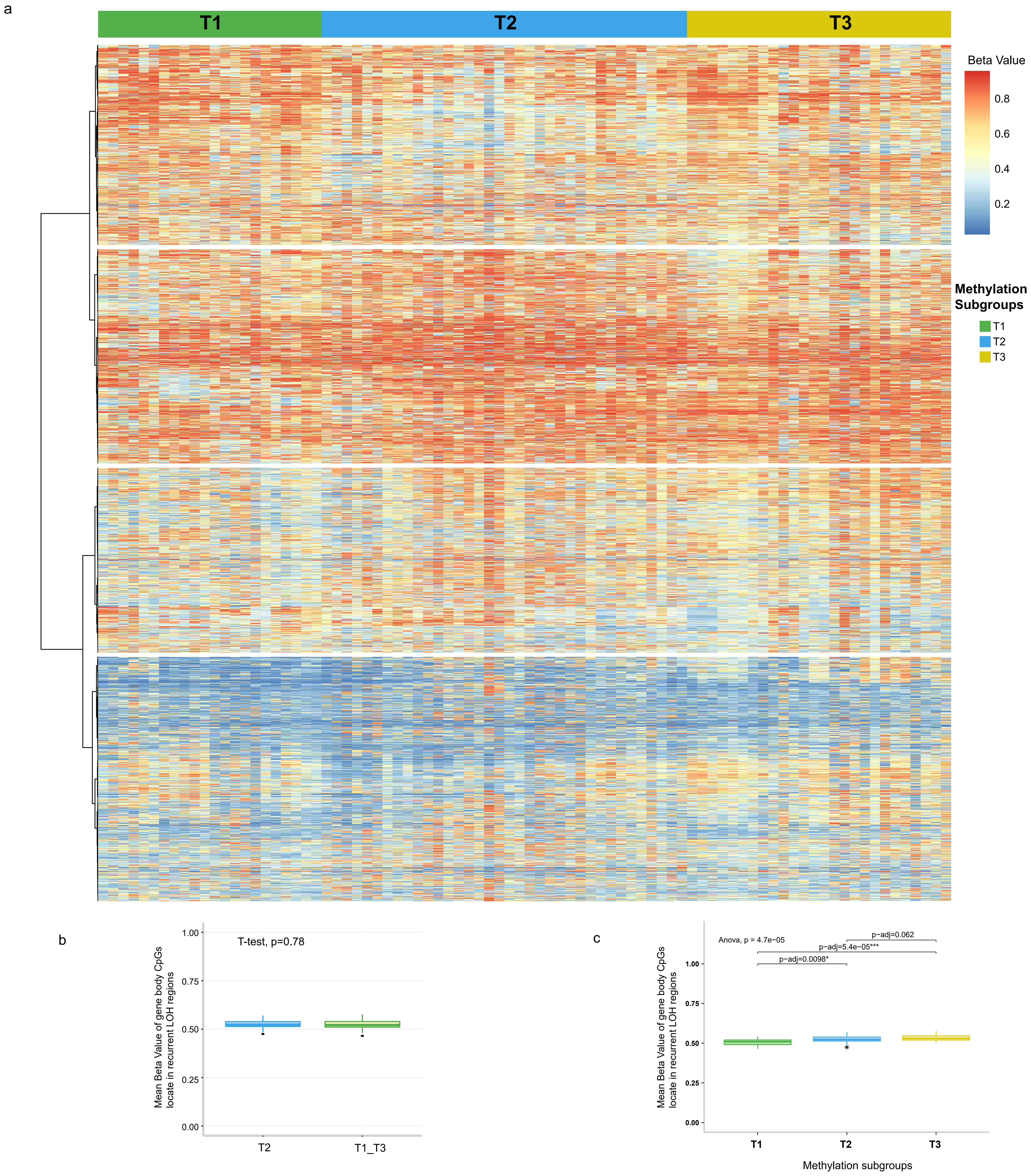

Supplementary Fig. 4: Gene body methylation across recurrent LOH regions. a) Heatmap of methylation levels of 7,316 most variable CpG sites (SD≥0.2 across tumors) located in the body of genes in chromosomes that presented recurrent LOH. b) Methylation levels do not suggest loss of methylation in subgroup T2 (n=36) compared to the other two subgroups together (n=48; p value=0.78; T test). c) T1 presented lower levels of methylation (n=22; adjusted p value=0.0098) than T2 (n=36) which includes tumors with recurrent LOH of half of the genome.

a

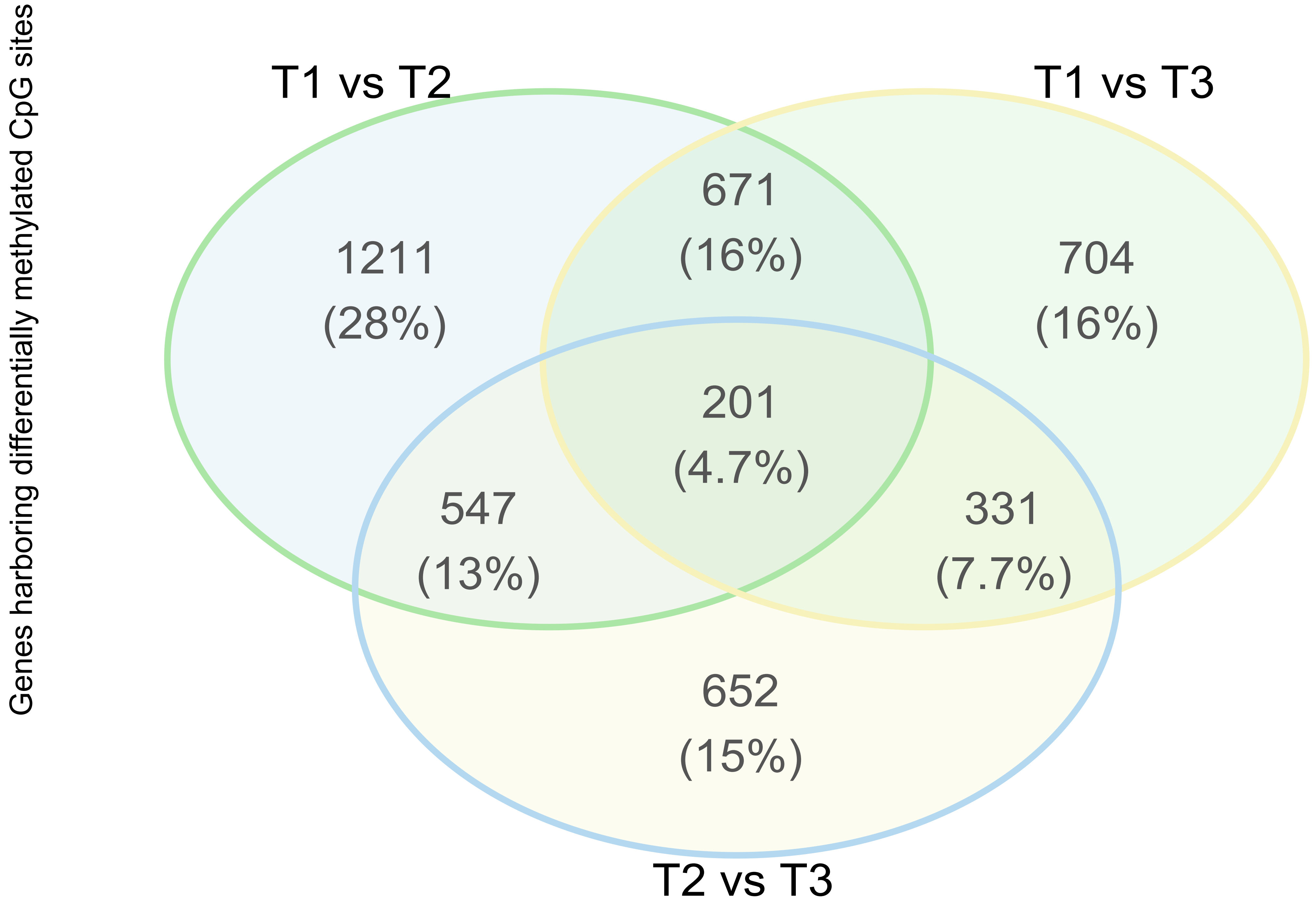

b

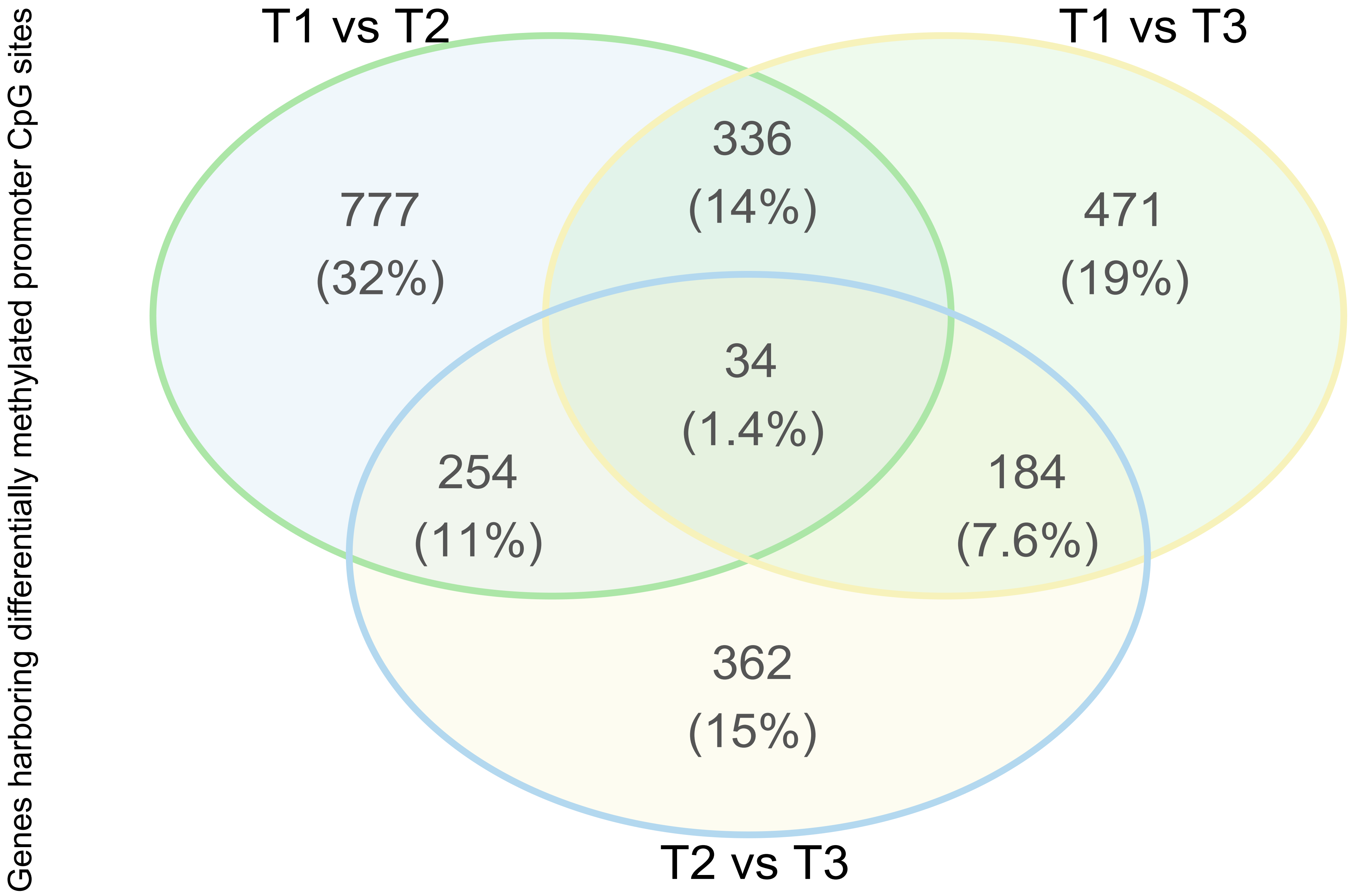

Supplementary Fig. 5: Number of genes with differentially methylated CpG sites. a) Number of genes harbouring CpG sites differentially methylated between methylation subgroups. b) Number of genes with promoter CpGs differentially methylated between subgroups. A total of 1,401 genes harboured differentially methylated promoter CpG sites between subgroups T1 and T2, 1,025 genes between T1 and T3 and 834 genes between T2 and T3.

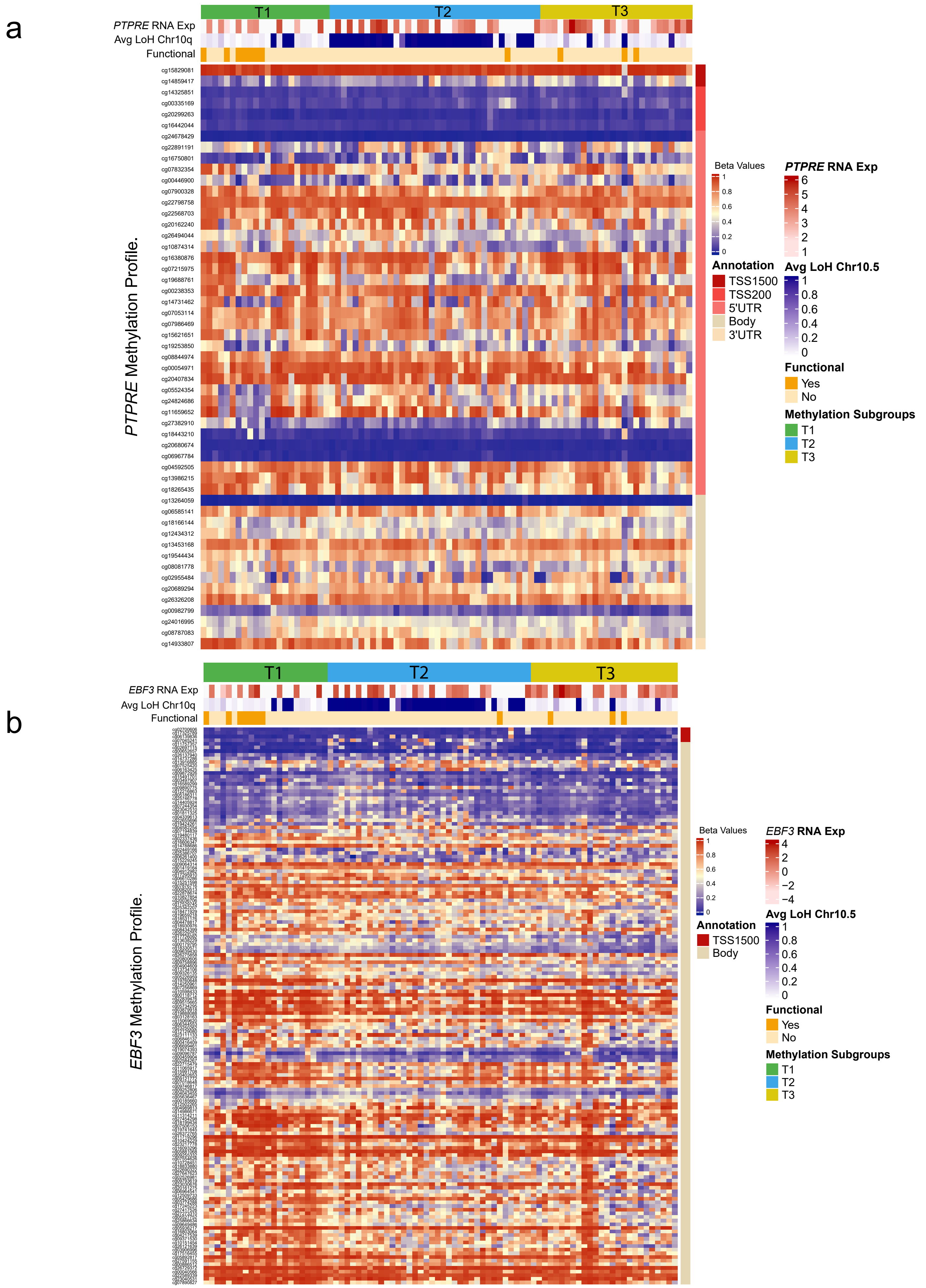

Supplementary Fig.6: Genes flanking *MGMT* gene. a) Methylation across *PTPRE* gene at the 5' of the transcription start of *MGMT*. b) Methylation across *EBF3* gene at the 3' end of *MGMT*. Tumors in T2 subgroup do not show patterns of hypo-methylation in the gene body observed in *MGMT* gene (see Fig.3).

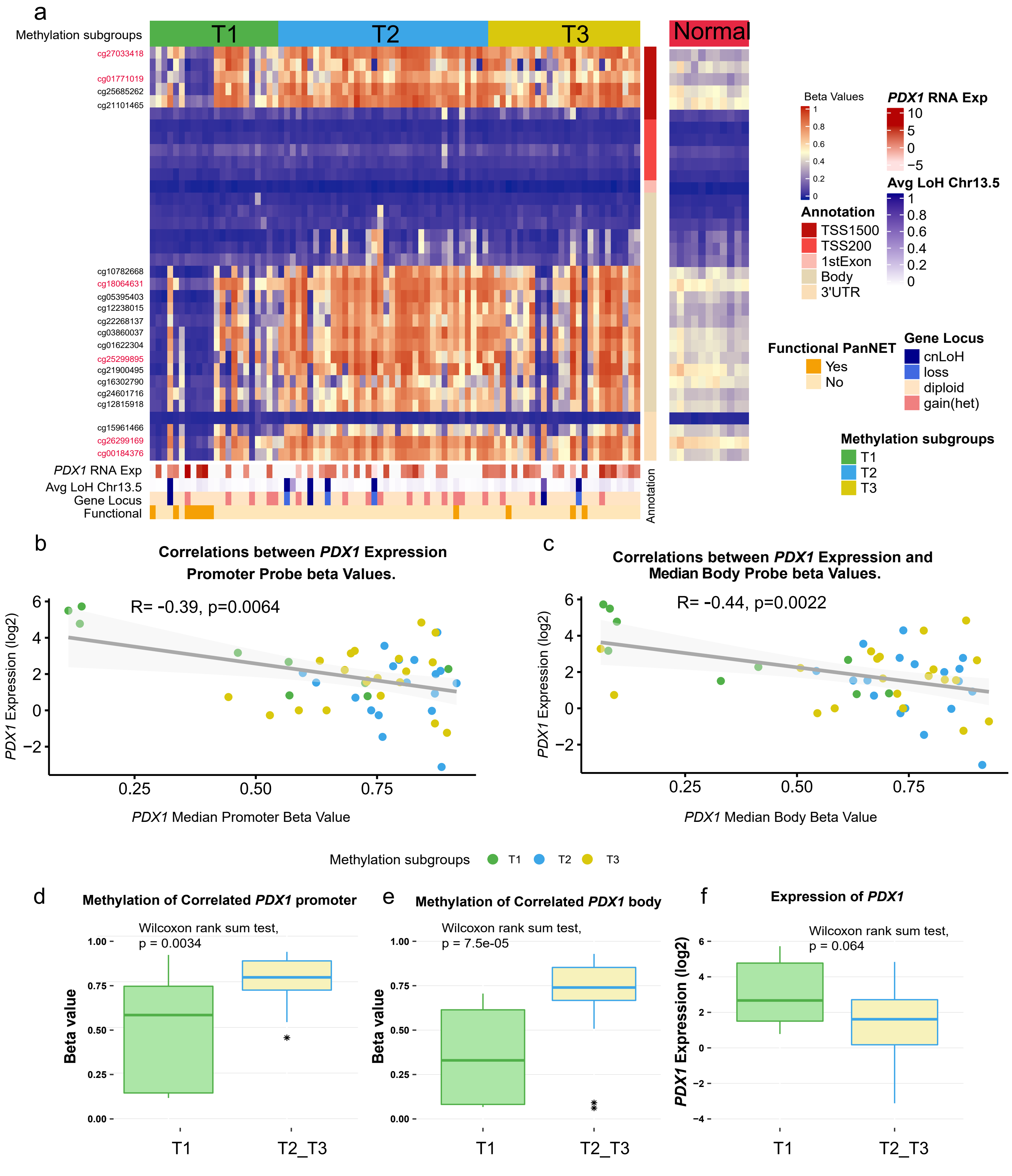

Supplementary Fig.7: *PDX1* gene methylation patterns. a) Heatmap of methylation levels of all CpG sites across the *PDX1* gene. The 19 CpG sites indicated on the left were differentially methylated between at least 2 subgroups, 6 of which (indicated in red) correlated with *PDX1* gene expression as assessed in 47 cases with RNASeq data between at least 2 subgroups (Supplementary Data 5). Levels of methylation in normal adjacent pancreata of CpG sites mapped to the *PDX1* gene is presented on the right. b) Correlation of promoter methylation (cg27033418) and gene expression. c) Correlation of gene body methylation (average of methylation levels of probes cg18064631 and cg25299895) and *PDX1* gene expression. d) Methylation level of promoter CpG correlated with gene expression in subgroup T1 (n=22) vs subgroups T2 and T3 (n=62). e) Methylation level of body CpGs correlated with gene expression in subgroup T1 (n=22) vs subgroups T2 and T3 (n=60). f) Expression levels of T1 tumors (n=9) and T2 and T3 tumors (n=38).

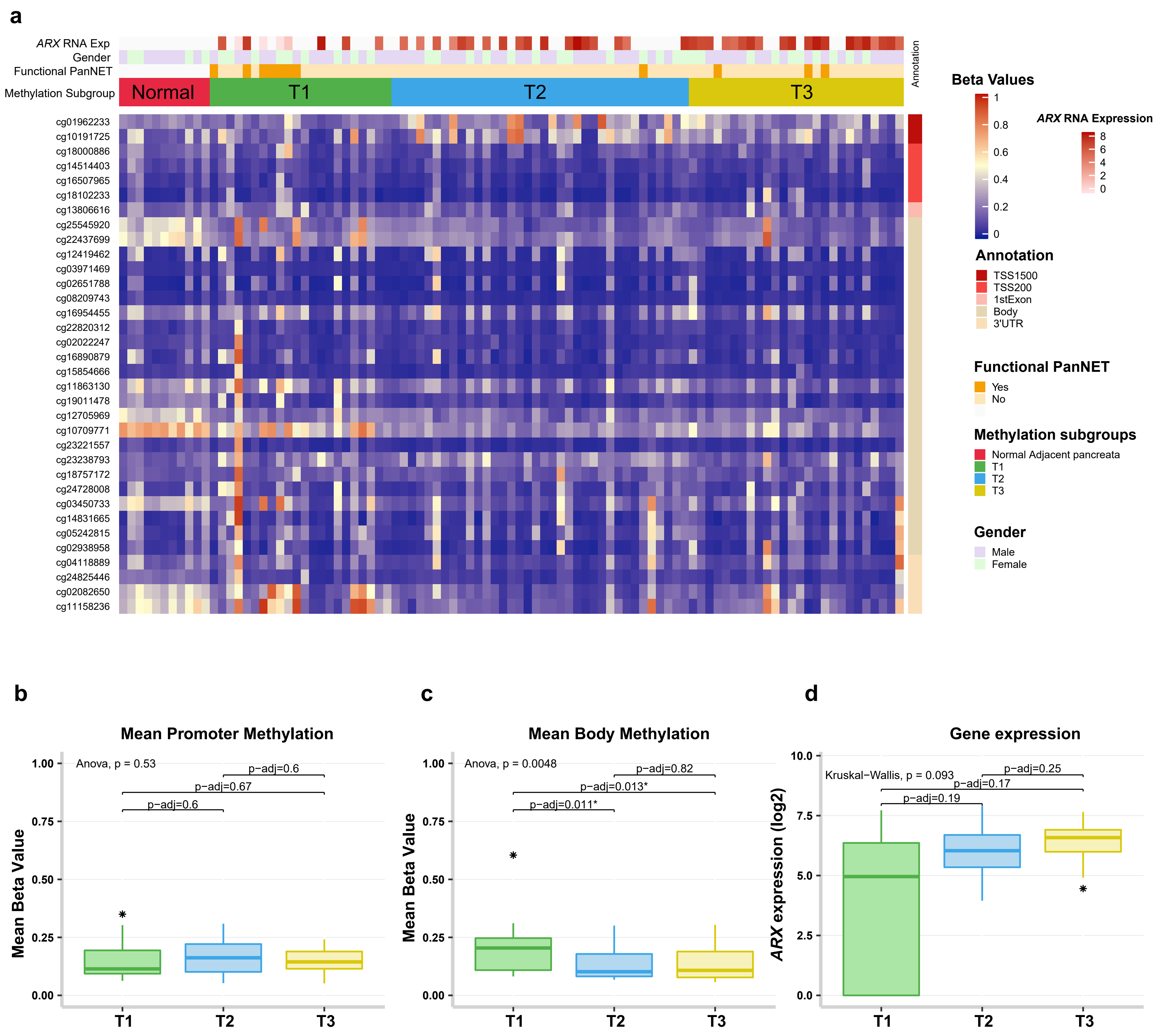

Supplementary Fig.8: Methylation and gene expresssion levels for *ARX* gene across subtypes. a) Heatmap of methylation levels of all CpG sites across the *ARX* gene across normal adjacent pancreata and tumours (columns). Tumours are presented in the order of cluster on Supplementary Fig.1. Females have higher level of methylation than males. b) Mean beta values of promoter methylation across subgroups. c) Mean beta values of body methylation acros subgroups. There is no difference in methylation across the groups.
d) *ARX* gene expression levels of T1 (n=9), T2 (n=18) and T3 tumors (n=20). The box within the boxplots represents a range of values from the first to third quantile and the line within represents the median value of the distribution. The whiskers represent the maximum and minimum values of the distribution excluding outliers and an asterisk represents any outlier.

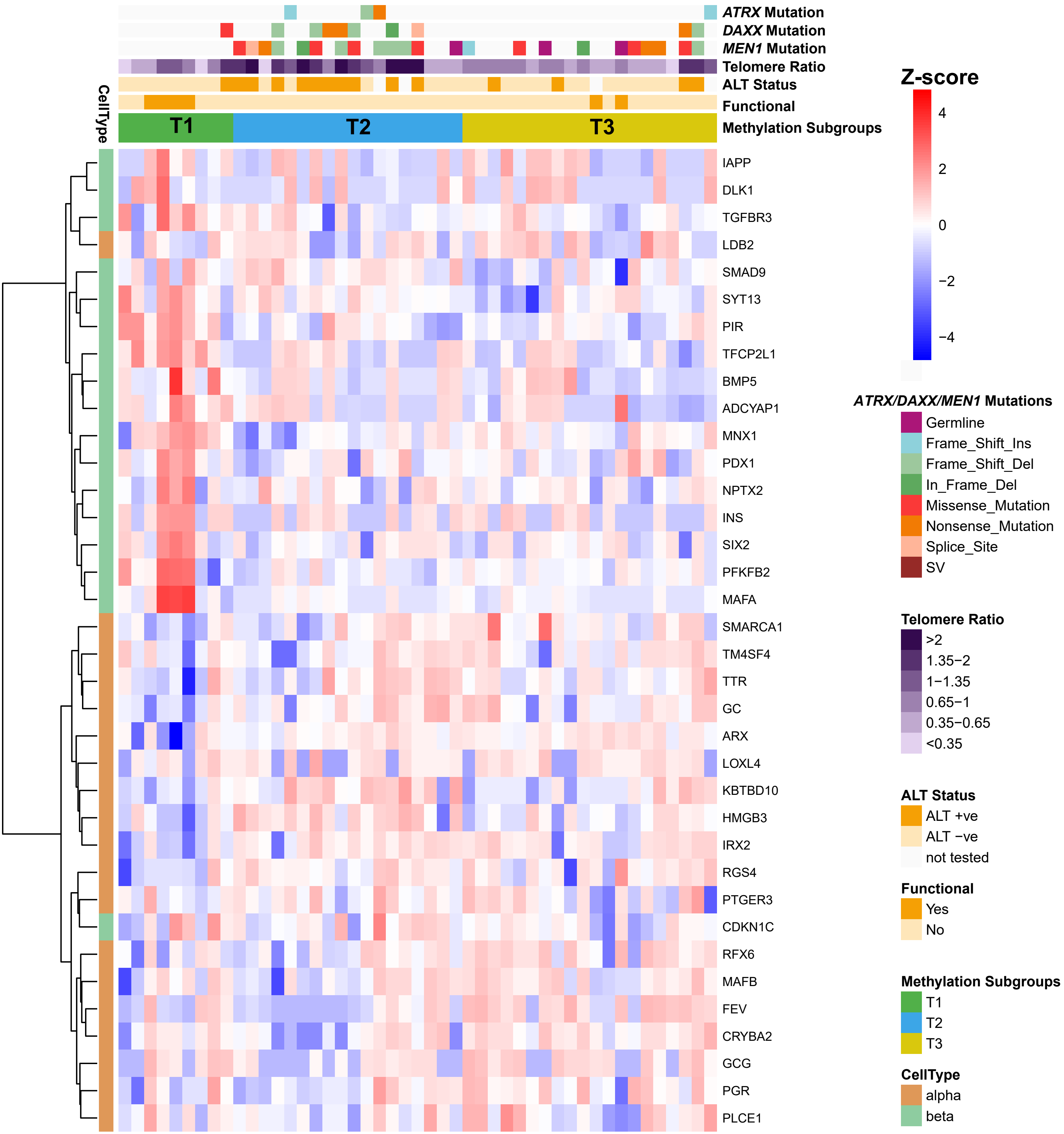

Supplementary Fig.9: Alpha and beta cells profile. Gene expression of gene markers for alpha and beta pancreatic cells for the 47 tumors with RNASeq data according to the gene list presented by Muraro et al.[19].

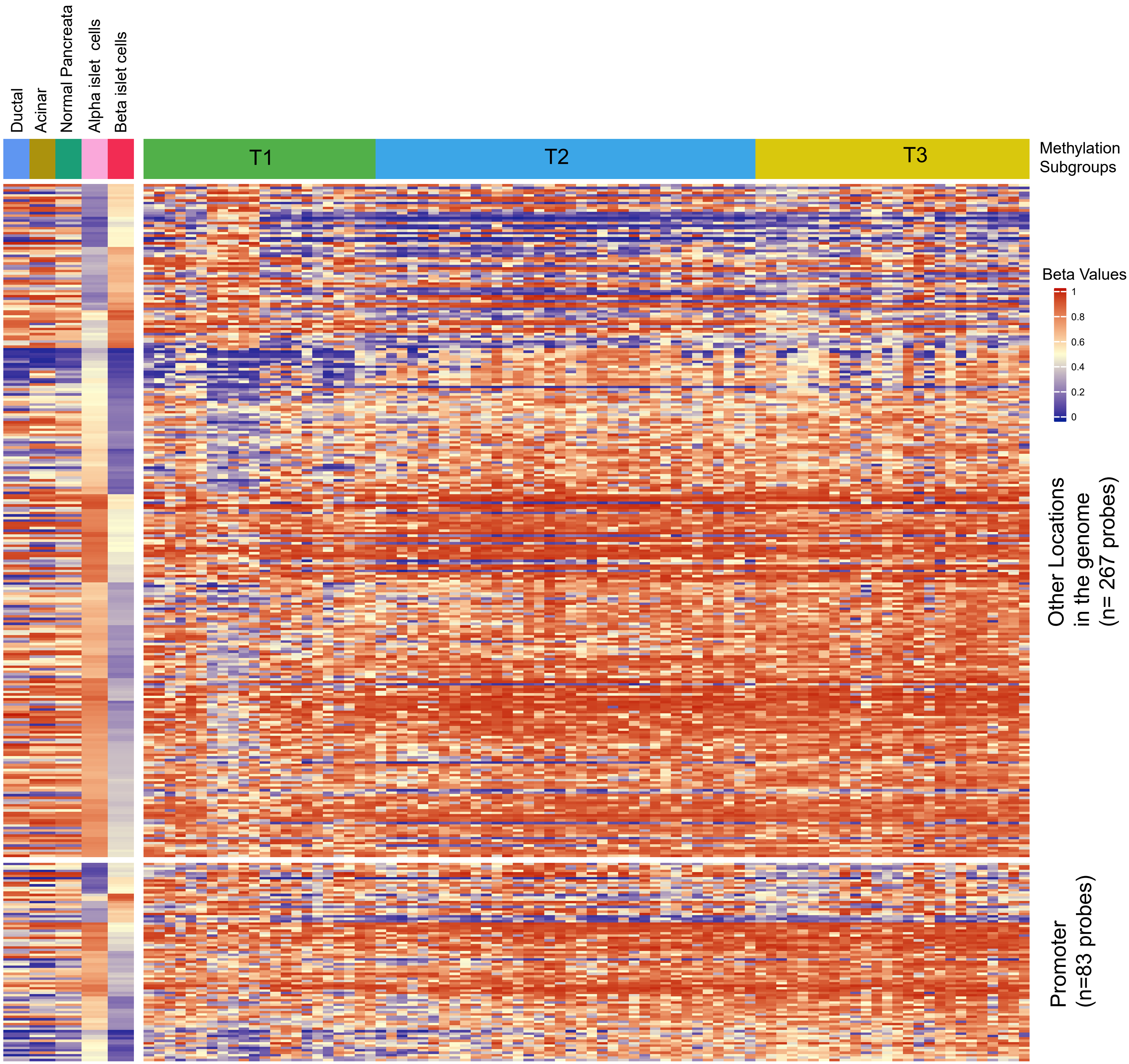

Supplementary Fig.10: Heat map of CpG sites differentially methylate in islet cells (2 alpha and 3 beta)[24] and tumours. Tumor (columns) are presented in the same order as clustering analysis Supplementary Fig.1. Probes (rows) were clustered between alpha and beta cells using Euclidean distance measures with Ward D clustering method, to highlight differenced between alpha and beta islet cells. Rows for other samples are plotted in the same order as alpha and beta islet cells. Probe selection for the heat map: Probes that were uniquely differentially methylated between islet (alpha and beta) and each tumour subgroup, these probes were further filtered, so only those that had a mean delta beta value between the alpha and beta islet cells >= 0.3 were retained. This selection was done to identify probes that most differentiated the two cell types and could give us insights about potential origin of tumour subgroups. The heatmap also shows methylation in ductal and acinar cells[24] and the average methylation of 11 adjacent pancreata.

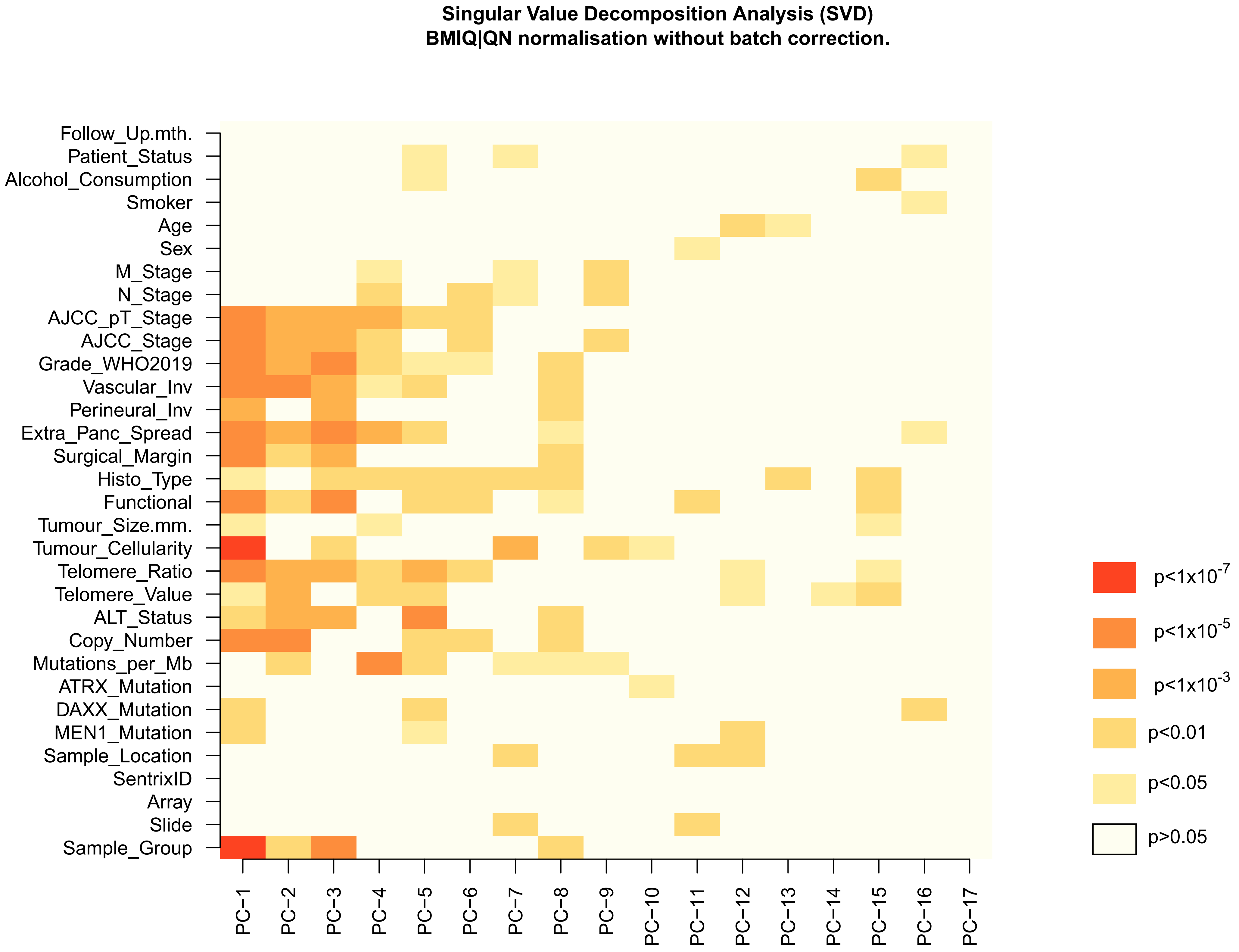

Supplementary Fig.11: Singular Value Decomposition Analysis for methylation data. No significant batch effect was observed with no significant variability in the 7 principal components. Sample Group (tumor and adjacent normal pancreata) as expected explain a significant proportion of the variability together with other genomic and clinical features of the cohort.

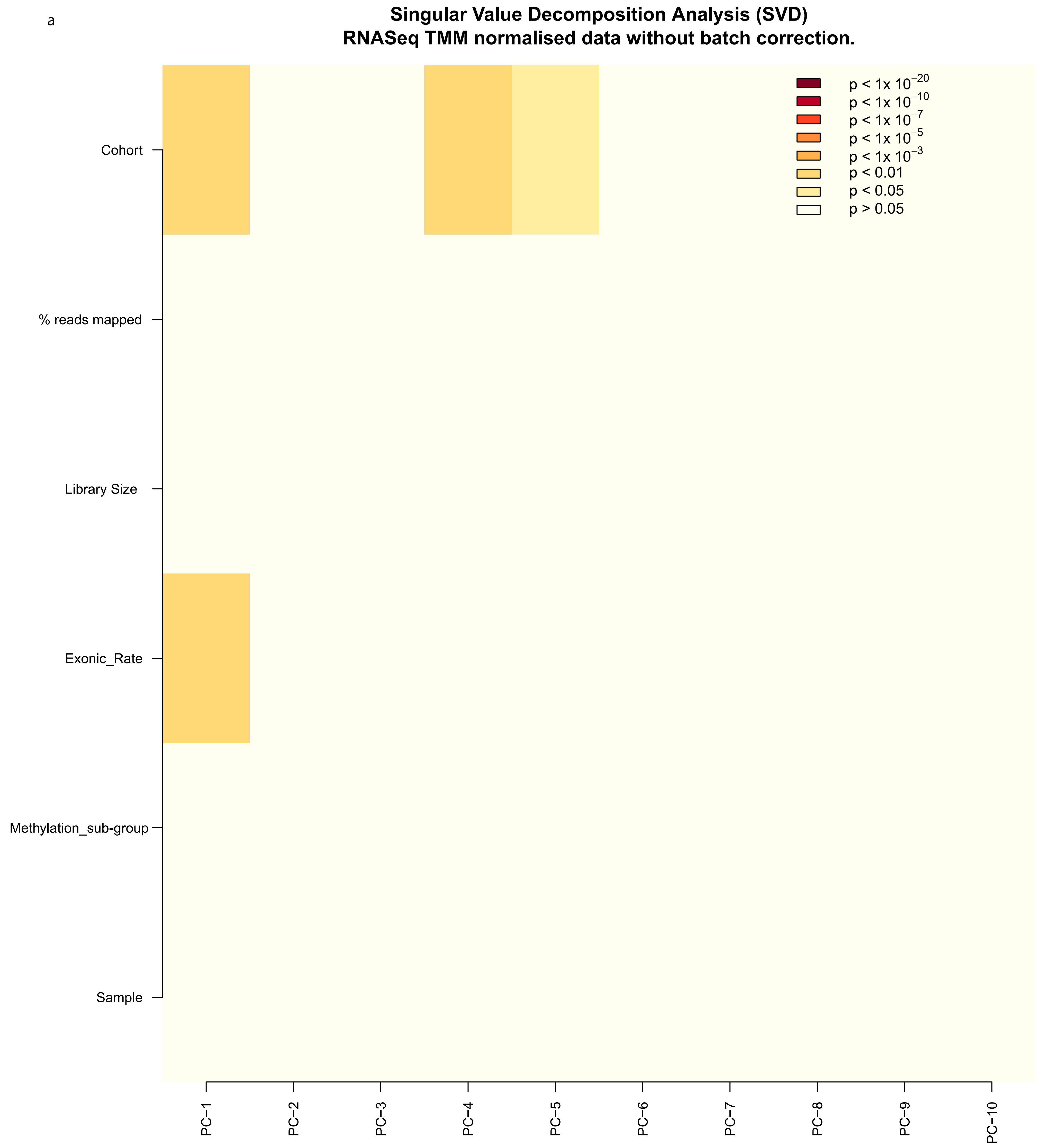

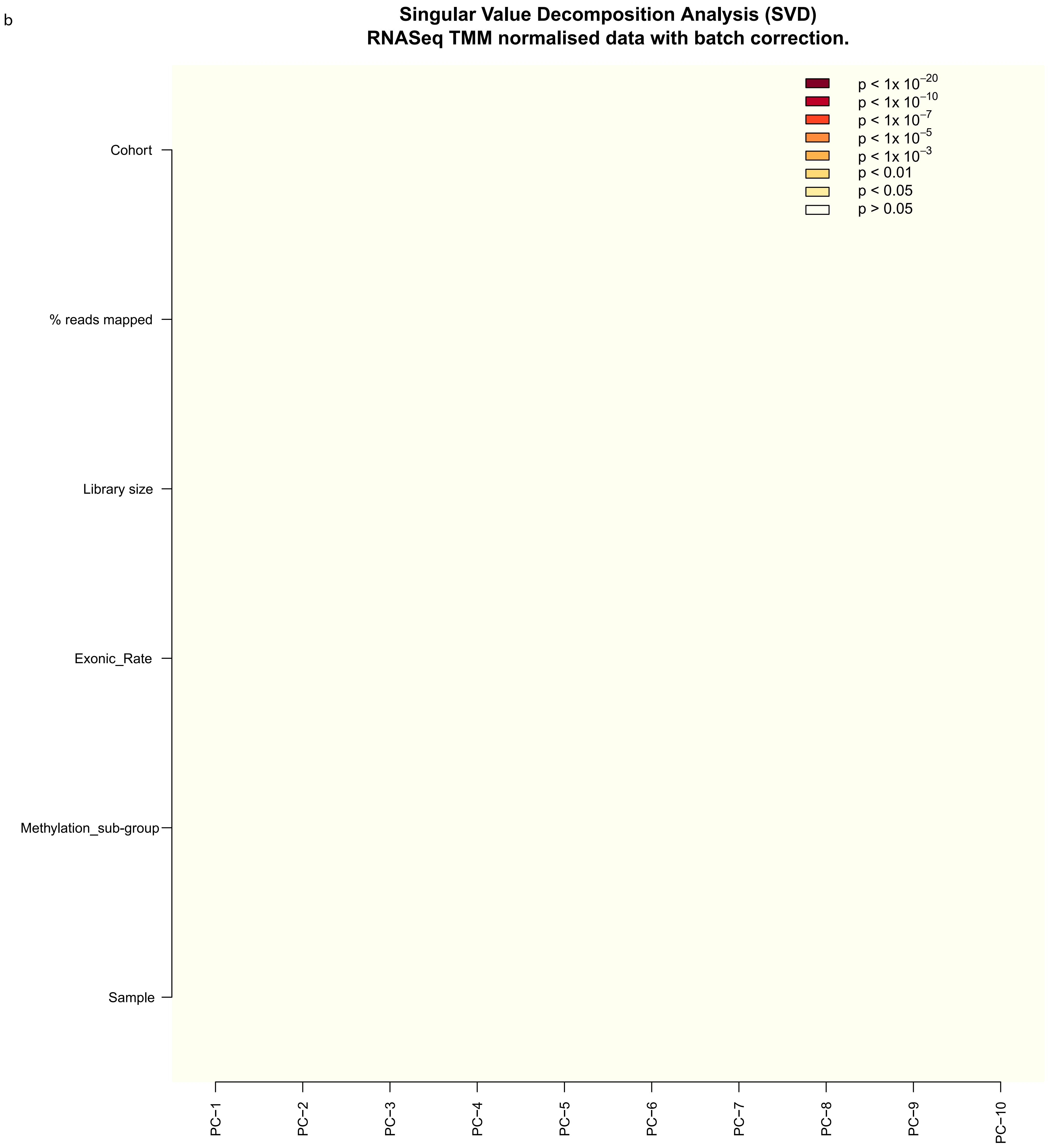

Supplementary Fig.12: RNASeq batch effect. a) Singular value Decomposition Analysis for RNASeq data. There was significant batch effect between the two sets of RNASeq used here. b) Singular value Decomposition Analysis after batch correction.

Supplementary Table 1. Number of probes removed from analysis. Study evaluated 411,159 probes.

Filter Process	# Probes Filtered	# Total Probes Filtered	Probes remaining	
**Imported Probes**			**485,512**	
Probes with detected p-value > 0.01	3,180	3,180	482,332	removed before initial QC and normalisation
Probes with fewer than 3 beads in >= 5% of samples	869	4,049	481,463	removed before initial QC and normalisation
**Probes normalised**			**481,463**	
Non CpG probes	3,066	7,115	478,397	
Probes on the X,Y chromosomes	10,103	17,218	468,294	
Probes with SNP-related polymorphisms	49,997	67,215	418,297	
Probes mapping to multiple regions	7,138	74,353	411,159	
