# Supplementary material for: DNA methylation patterns identify subgroups of pancreatic neuroendocrine tumors with clinical association
Source: Commun Biol. 2021 Feb 3;4:155. doi: 10.1038/s42003-020-01469-0 (PMC7859232; doi:10.1038/s42003-020-01469-0)
Supplement: Supplementary file 2 — Description of Additional Supplementary Files [file 42003_2020_1469_MOESM2_ESM.docx]

Description of Additional Supplementary Files

Suppl. Data 1 - Clinical and genomic information of the cohort.

Suppl. Data 2 - Probes differentially methylated between sub-groups T1 and T2.

Suppl. Data 3 - Probes differentially methylated between sub-groups T1 and T3.

Suppl. Data 4 - Probes differentially methylated between sub-groups T2 and T3.

Suppl. Data 5 - Correlation of differentially methylated sites and gene expression.

Suppl. Data 6 - Genes that harbour 5 or more probes differentially methylated between subgroups

Suppl. Data 7 - ARX gene methylation and gene expression

Supplementary_Data_8 (source data for figures)
